# The Clinical Risk Factors of Adenovirus Pneumonia in Children Based on the Logistic Regression Model: Correlation with Lactate Dehydrogenase

**DOI:** 10.1155/2022/3001013

**Published:** 2022-03-24

**Authors:** Yuqiao Liu, Yang Shen, Botao Wei

**Affiliations:** Department of Infectious Diseases, Tianjin Children's Hospital, 238 Longyan Road, Beichen District, Tianjin, China

## Abstract

**Methods:**

Children with bacterial pneumonia (41 cases) and adenovirus pneumonia (179 cases) hospitalized in Tianjin Children's Hospital from January to October 2020 were selected. The differences in baseline and clinical characteristics between children with two pneumonias, respectively, were compared via the chi-square test and Wilcox test. The Least Absolute Shrinkage and Selection Operator (LASSO) model was applied to screen the pneumonia type-related characteristics. Patients were randomly divided into the training set (*n* = 154) and test set (*n* = 66). The logistic model was constructed using the screened characteristics in the training set to predict whether the cases are bacterial pneumonia or adenovirus pneumonia. Finally, the model was validated by receiver operating characteristic (ROC) curve and area under curve (AUC) in the test set.

**Results:**

The age (*p* < 0.001), hospital stay (*p* < 0.001), tonsil condition (*p* < 0.001), interleukin-6 (IL-6; *p*=0.033), and lactate dehydrogenase (LDH; *p* < 0.001) between children with bacterial pneumonia and adenovirus pneumonia were significantly different. Sex, tonsil condition, age, hospital stay, r-glutamyltransferase (r-GT), and LDH levels were the factors associated with the types of pneumonia. Compared with bacterial pneumonia, children with adenovirus pneumonia were younger (OR = 0.207, 95% CI: 0.041–0.475), with longer hospital stay (OR = 7.974, 95% CI: 2.626–74.354) and higher LDH expression level (OR = 1.025, 95% CI: 1.006–1.060). 92.4% types of pneumonia were correctly predicted, and the AUC value of the model was 0.981.

**Conclusion:**

The LDH level was the associated factor to predict the types of pneumonia. Adenovirus pneumonia was associated with earlier age and longer hospital stay than bacterial pneumonia. The established model can well predict the types of pneumonia in children and provide clinical basis for guiding the individualized treatment of children.

## 1. Introduction

As a severe viral pneumonia, adenovirus pneumonia is mostly seen in children aged from 6 months to 2 years [1]. At the beginning of the disease, children often have persistent fever. After 3–5 days, children patients are likely to present dyspnea and systemic poison, some of whom suffered from diarrhea, vomiting, and even severe abdominal distension symptoms, while a small number of them suffered from conjunctival congestion and tonsil secretions [2]. Children at young age, with underlying disease or recurrent respiratory tract infection, are prone to severe case [3]. In addition, human adenovirus load and fever time are risk factors for pneumonia severity [4].

Lactate dehydrogenase (LDH) is an important enzyme involved in the anaerobic metabolism and ubiquitously present in almost all cells in the body, which responds to tissue damage in a nonspecific manner [5]. Serum LDH level may elevate due to hemolysis, cancer, and human immunodeficiency virus infection [6]. According to its characteristics, many studies reported the role of LDH in assessing the clinical severity of coronavirus disease 2019 (COVID-19) [7]. Moreover, the inhibition of LDH can reduce the conversion of glucose to lactate, thus reversing the Warburg effect to deprive cancer cells of their ability to survive in the hypoxic tumor microenvironment [8].

Least absolute shrinkage and selection operator (LASSO) is an effective embedded method of feature selection, which minimizes the sum of squares of residuals and adds regularized penalty term (*L*_1_) to estimate coefficients and screen variables simultaneously [9]. The model is a ridge regression model when the penalty term is *L*_2_, and elastic net integrates the penalty terms *L*_1_ and *L*_2_. K. Aheto et al. [10] applied three models to predict malaria prevalence, and these models screen 11, 15, and 13 characteristics out of 15 characteristics, respectively, with LASSO showing the smallest prediction error.

We constructed the prognostic model based on machine learning to evaluate the risk factors for adenovirus pneumonia and then validated the accuracy of this model.

## 2. Materials and Methods

### 2.1. Objects

This study selected 41 children with bacterial pneumonia and 179 children with adenovirus pneumonia who were hospitalized in Tianjin Children's Hospital from January to October 2020. The exclusion criteria were as follows: children were less than 14 years old, and children were diagnosed with adenovirus pneumonia according to Guidelines for Diagnosis and Treatment of Adenovirus Pneumonia in Children (2019 Version) [2]. The study was approved by the Ethics Committee of Tianjin Children's Hospital (L2021-19). As the study was a retrospective study and no clear information of patients appeared in the study, the informed consent was waived.

### 2.2. Data Collection

The baseline characteristics of children were collected, including age, sex, hospital stay, tonsil enlargement, conjunctival congestion, alanine aminotransferase (ALT), aspartate aminotransferase (AST), r-glutamyltransferase (r-GT), interleukin-6 (IL-6), LDH, CD4 T cell proportion, and CD8 T cell proportion.

### 2.3. Statistical Methods

**Figure 1:**
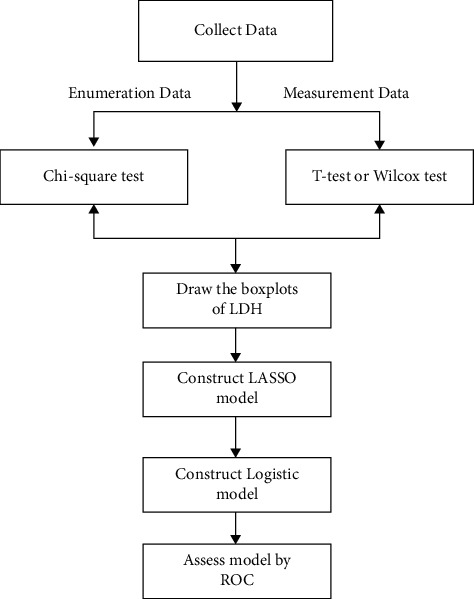
The flowchart of the statistical analysis.

The chi-square test was utilized to analyze the differences of enumeration data. The Shapiro–Wilk test was applied to detect the normality of measurement data. The *t*-test was chosen if the data satisfied the normal distribution, and the Wilcox test was employed or otherwise. The differences in LDH levels between children with adenovirus and bacterial pneumonia were compared via boxplots. The LASSO model and logistic model were utilized by the R software “glmnet” package. ROC curve drawing and AUC value calculation were based on the “plotROC” package and “pROC” package. *P* value < 0.05 was considered statistically significant (Figure 1).

## 3. Results

### 3.1. Analysis of Differences

The differences of enumeration data between two groups, including sex, tonsil condition, and conjunctiva congestion, were analyzed by the chi-square test. No significant differences were found in the age and conjunctiva congestion (*p*=0.072, *p*=0.208), while the differences in tonsil condition were statistically significant (*p* < 0.001). Children with adenovirus pneumonia were susceptible to tonsil enlargement.

**Figure 2:**
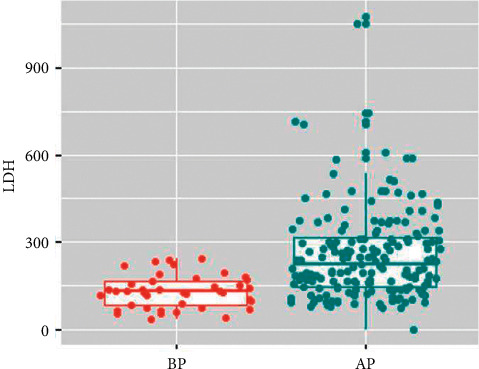
Comparison of LDH between children with bacterial and adenovirus pneumonia. BP, bacterial pneumonia; AP, adenovirus pneumonia.

Shapiro–Wilk confirmed that all measurement data did not fit the normal distribution. Thus, the Wilcox test was applied to compare the differences between the groups, as given in Table 1. The age (*p* < 0.001), hospital stay (*p* < 0.001), tonsil condition (*p* < 0.001), IL-6 (*p*=0.033), and LDH (*p* < 0.001) between two groups were significantly different. Children with adenovirus pneumonia were younger and had longer hospital stay and higher IL-6 and LDH levels. The results of boxplot (Figure 2) indicated that LDH level in children with adenovirus pneumonia was higher.

### 3.2. Construction of the LASSO Model

**Figure 3:**
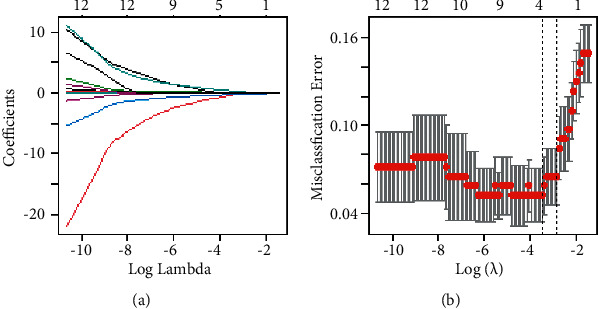
The results of LASSO regression. (a) The characteristic coefficient varies with the parameter. (b) Classification errors vary with parameter in the 10-fold cross-validation.

The LASSO model (Figure 3(a)) was constructed to analyze the characteristics that affected pneumonia types. 10-fold cross-validation was applied to select the corresponding model parameters as the classification error was minimum. In this model, the classification effect of the model was optimal when 6 characteristics were reserved (Figure 3(b), Table 2), that is, the types of pneumonia were related to sex, tonsil condition, age, hospital stay, r-GT, and LDH levels.

### 3.3. Analysis of the Logistic Regression Model

Children were randomized to the training set (*n* = 154) and test set (*n* = 66). The binary logistic model was constructed based on the 6 screened characteristics in the training set to predict the classification effect (Table 3). Age (*p*=0.008), hospital stay (*p*=0.010), and LDH (*p*=0.048) were statistically significant. According to odds ratio (OR), children with adenovirus pneumonia were younger (OR = 0.207, 95% CI: 0.041–0.475), with longer hospital stay (OR = 7.974, 95% CI: 2.626–74.354) and higher LDH level (OR = 1.025, 95% CI: 1.006–1.060), compared to bacterial pneumonia.

### 3.4. Model Assessment

**Figure 4:**
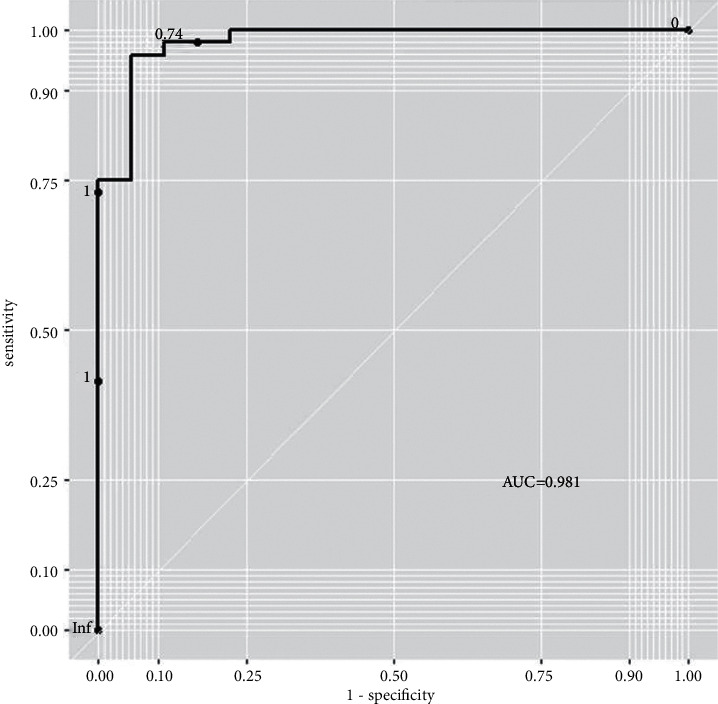
ROC of the logistic model.

The model was validated in the test set. According to the results of logistic regression, the type of pneumonia was predicted with 0.5 as the boundary. It was predicted as adenovirus pneumonia when the probability value of adenovirus pneumonia was bigger than 0.5; otherwise, it was bacterial pneumonia. The probability of correct classification of the model was 92.4%. ROC of the model was drawn (Figure 4), and AUC was 0.981, indicating that the model had high accuracy.

## 4. Discussion

The relevant characteristics of pneumonia types were analyzed in this study. Differential analysis confirmed that the age, hospital stay, tonsil condition, IL-6, and LDH levels were significantly different between children with bacterial and adenovirus pneumonias. LASSO was applied to screen the characteristics associated with pneumonia types, including sex, tonsil condition, age, hospital stay, r-GT, and LDH levels. The constructed logistic regression model showed that age, hospital stay, and LDH levels were statistically significant. Children with adenovirus pneumonia were younger and had longer hospital stay compared with children with bacterial pneumonia. AUC value of the model was 0.981, and the model had high accuracy. Yang et al. screened predictors for adenovirus pneumonia applying LASSO regression and developed nomogram, followed by the evaluation of nomogram discrimination via ROC curves, whose AUC value is 0.79 [11]. Whereas, the prediction model constructed in our study had higher accuracy, and we subsequently explored the correlation between LDH level and adenovirus pneumonia in children.

LDH level can reflect lung conditions [12], and serum LDH elevates in patients with severe adenovirus tract infection [13]. In this study, the significance of LDH level was confirmed in the process of statistical analysis, characteristics screening, and regression model construction. The results indicated that LDH was the relevant factor of adenovirus pneumonia, and children with adenovirus pneumonia had higher LDH expression, which was consistent with the results of Liu et al. [14]. In addition to indicating the existence of viral pneumonia, LDH level can also reflect the severity of pneumonia [15]. Liu et al. [14] and Wu et al. [16] demonstrated that LDH level was higher in patients with severe adenovirus tract infection.

Age and hospital stay were different in this study, and children with adenovirus pneumonia were younger and had longer hospital stay. The characteristics of young age of adenovirus patients were reflected in many studies. The Guideline of Diagnosis and Treatment of Adenovirus in Children pointed out that adenovirus pneumonia is mostly seen in children aged from 6 months to 5 years [2]. Infants under 6 months have immune ability due to maternal antibodies and develop their own antibodies as they grow, reducing the risk of adenovirus infection [17]. The longer hospital stay in children with adenovirus pneumonia may result from its severity. Adenovirus pneumonia has high mortality [18] and leads to multisystem complications [19]. Kuo et al. [20] found no difference in age and hospital stay between children with adenovirus and nonadenovirus, and the different results may be explained by the small number of samples (*n* = 48) included in their study.

The CD4/CD8 ratio varies with the immune dysregulation caused by viral infection [21]. But no significant difference was found between children with adenovirus and bacterial pneumonia in CD4, CD8, and CD4/CD8 levels. Mei et al. [22] studied children with viral pneumonia and found that children with viral pneumonia have higher CD4 and CD4/CD8 levels and lower CD8 levels compared to normal children.

In conclusion, it was found that LDH level might be a predictor of adenovirus pneumonia in children, which provides an idea to distinguish adenovirus pneumonia from bacterial pneumonia. Meanwhile, the analyses of age, hospital stay, tonsil condition, and IL-6 reveal the clinical characteristics of adenovirus pneumonia. However, only one model was used to screen the relevant factors of adenovirus pneumonia, and it will be more reliable to apply multiple models and compare the results to screen characteristics. The inclusion of more pneumonia types in future studies may enable LDH data to be more clinically useful.

## Figures and Tables

**Table 1:** Baseline and pathological characteristics of children.

Characteristics	BP^a^ (*N* = 41)	AP^b^ (*N* = 179)	*P* value
Sex, %			0.072
Male	26 (63.4)	83 (46.4)	
Female	15 (36.6)	96 (53.6)	
Age (years old)	13.000 (7.000, 14.000)	3.000 (1.000, 5.000)	<0.001
Length of stay (days)	3.000 (1.000, 5.000)	6.000 (4.000, 8.000)	<0.001
Tonsil, %			<0.001
Normal	14 (34.1)	146 (81.6)	
Enlargement	27 (65.9)	33 (18.4)	
Conjunctiva, %			0.208
Normal	40 (97.6)	161 (89.9)	
Hyperemia	1 (2.4)	18 (10.1)	
ALT, U/L	13.000 (11.000, 19.000)	13.000 (11.000, 16.000)	0.415
AST, U/L	38.000 (29.000, 42.000)	35.000 (26.500, 45.000)	0.669
r-GT, U/L	10.000 (9.000, 14.000)	10.000 (8.000, 13.000)	0.477
IL-6, pg/mL	11.820 (3.760, 37.390)	28.980 (7.825, 51.100)	0.033
LDH, U/L	321.000 (276.000, 356.000)	415.000 (335.000, 508.000)	<0.001
CD4, %	34.810 (32.090, 39.280)	34.800 (29.225, 40.940)	0.555
CD8, %	23.290 (21.380, 26.650)	22.890 (18.645, 27.900)	0.264
CD4/CD8^c^	1.440 (1.310, 1.790)	1.500 (1.180, 2.050)	0.744

^a^BP, bacterial pneumonia; ^b^AP, adenovirus pneumonia; CD4/CD8, the ratio of CD4 cells to CD8 cells.

**Table 2:** The optimal retention parameters and corresponding coefficients verified by 10-fold cross-validation.

Characteristics	Coefficient
Sex	0.062
Tonsil condition	−1.237
Age	−0.388
Length of stay	0.384
r-GT	−0.018
LDH	0.003

CD4/CD8, the ratio of CD4 cells to CD8 cells.

**Table 3:** The results of logistic regression.

Characteristics	B	Wald	OR (95% CI)	*P*
Sex	3.103	2.854	22.268 (1.252, 3570.801)	0.091
Tonsil condition	−4.101	2.971	0.017 (0, 0.669)	0.085
Age	−1.575	7.029	0.207 (0.041, 0.475)	0.008
Length of stay	2.076	6.677	7.974 (2.626, 74.354)	0.010
r-GT	−0.063	2.202	0.939 (0.856, 1.054)	0.138
LDH	0.025	3.905	1.025 (1.006, 1.060)	0.048

## Data Availability

No data were used to support this study.
